# Toppgene筛选肺腺癌候选疾病基因

**DOI:** 10.3779/j.issn.1009-3419.2010.04.02

**Published:** 2010-04-20

**Authors:** 桂平 王, 云 叶, 文岭 郑, 文丽 马

**Affiliations:** 1 510515 广州，南方医科大学基因工程研究所 Institute of Genetic Engineering, Southern Medical University, Guangzhou 510515, China; 2 510180 广州，广州医学院护理学院 Nurse School, Guangzhou Medical College, Guangzhou 510180, China; 3 545006 柳州，广西工学院生物与化学工程系 Department of Biological and Chemical Engineering, Guangxi University of Technology, Liuzhou 545006, China

**Keywords:** Toppgene, 基因功能相似性, 肺腺癌, 基因, Toppgene, Gene function similarity, Lung adenocacinoma, Gene

## Abstract

**背景与目的:**

肺腺癌是危害人类健康最主要的肺癌类型之一，其发生机制仍不清楚。本研究利用生物信息学方法，筛选新的肺腺癌候选基因，为揭示肺腺癌发病机制提供依据。

**方法:**

从GEO数据库中获得GSE10072和GSE7670两个数据集，然后利用dchip软件进行差异表达基因分析，将其获得的差异基因定义为“检测基因集”（test gene set）；采用genecard和Fable文献挖掘已知肺腺癌疾病基因，并将其定义为“训练基因集”（train gene set）；最后，利用Toppgene筛选肺腺癌候选基因，并通过荧光定量PCR对其获得的部分基因进行验证。

**结果:**

获得一个含344个基因的“检测基因集”和含277个基因的“训练基因集”。采用Toppgene共获得36个候选疾病基因，其中21个基因则在肿瘤方面的研究几无报道。荧光定量PCR实验研究发现，*CD36*、*PMAIP1*及*FABP4*三个基因在A549细胞中均为下调表达，与芯片数据一致。

**结论:**

Toppgene可发现新的肺腺癌候选疾病基因，为下一步发现特异性肺腺癌致病基因提供理论依据。

## Abstract (en)

**Background and objective:**

Lung adenocarcinoma (AC) is the most common type of lung cancer, however, its mechanism of oncongenesis is still unknown. The aim of this study is to screen candidate genes of lung adenocarcinoma using bioinformatics strategy and elucidate the mechanism of lung adenocarcinoma.

**Methods:**

Two published microarray data (GSE7670 and GSE10072) was obtained from Gene Expression Omnibus (GEO). Significance analysis of microarrays was performed with the software dchip, and differential expression genes from dchip analysis were defined as "test gene set". Genes correlated with lung adenocarcinoma, obtained by data mining tools genecard and Fable were regarded as "train gene set". Finally, candidate genes of lung adenocarcinoma were screened by the tool "Toppgene".

**Results:**

Three hundred and fortyfour differential genes were defined as "test gene set", and 277 genes correlated with lung adenocarcinoma were regarded as "train gene set". Thirty-six candidate genes were screened out by Toppgene, among them, 21 genes had nearly no report in cancer. In the following QRT-PCR experiment, *CD36*, *PMAIP1* and *FABP4* were down-regulated expression in A549, which coincided with the gene chip.

**Conclusion:**

It is demonstrated that Toppgene is useful in identification of the candidate genes of lung adenocacinoma, which provides the proof for the discovery of the specific disease genes.

肺癌是我国男性和女性最主要致死性癌症之一，包括小细胞肺癌和非小细胞肺癌^[1]^。肺腺癌（lung adenocarcinoma）属于非小细胞肺癌，是最常见的肺癌之一，发病率约占原发性肺癌的20%-30%，在许多国家腺癌已超过鳞状细胞癌。目前，人类对肺腺癌的发生机制仍不清楚，其发生发展可能与体内多种癌基因或抑癌基因的表达改变有关，如*k-ras*、*p53*、*p16Ink4*、*HER2*/*Ne*u和*COX-2*等。因此，发现新的肺腺癌致病基因，对于揭示肺腺癌发病机制及寻找新的药物治疗靶点有着重要意义。

目前，疾病基因发现的方法包括连锁分析法、基因序列相似性、基因功能相似性及蛋白质相互作用网络等多种途径，其中以基于基因功能相似性方法在人类疾病候选基因发现中的应用最广泛^[2-7]^。近年来，许多基于功能相似性的生物信息学方法在人类疾病基因发现发挥重要作用，加速人类疾病基因发现过程，如POCUS、PROSPECTR、SUSPECTS及Toppgene等，其中Toppgene具有高通量、快速、重复性好的优点，特别是可对基因提供更全面的评价^[2, 7, 8]^。为发现新的肺腺癌致病基因，本研究从GEO数据库中获取肺腺癌数据集，并进行差异基因分析，将获取的差异基因作为“检测基因集”；同时，采用genecard和Fable文献挖掘已知肺腺癌疾病基因，并将其定义为“训练基因集”；最后，利用Toppgene筛选肺腺癌候选基因，并通过荧光定量PCR对其获得的基因进行验证。

## 1 材料与方法

### 1.1 材料

Trizol RNA抽提试剂、PrimeScript^TM^逆转录试剂盒、SYBRPremix Ex Ta^TM^荧光定量PCR试剂盒均由中山医达安基因公司提供。3900台式高通量DNA合成仪、9700 PCR仪、7500全自动荧光定量PCR仪均为ABI产品。肺腺癌细胞株A549和人支气管上皮细胞16HBE由广州医学院医学实验中心提供，培养于含10%胎牛血清（FBS，杭州四季青）、双抗（青霉素100 U/mL、链霉素100 U/mL）的RPMI-1640培养基中。

### 1.2 方法

#### 1.2.1 获取GEO数据集

首先，我们从NCBI的GEO数据库（http:www.ncbi.nlm.nih.gov/geo）中下载2个基因表达谱数据集，即GSE7670和GSE10072。其中，GSE7670数据集来源于台湾台北荣民总医院（Taipei veterans general hospital），采用GPL96芯片平台（[HG-U133A] Affymetrix Human Genome U133A Array），包括27个配对的正常肺组织与肺腺癌组织、2个混合组织、2个商业化的正常肺组织、1个正常肺上皮细胞株与7个商业化肺癌细胞株，共64个样本；而另一个数据集GSE10072则来源于美国N.I.H遗传流行病学部（Genetic Epidemiology Branch），也采用GPL96芯片平台，疾病组织类型为肺腺癌，包括58个腺癌和49个正常肺组织，共107个样本。

#### 1.2.2 肺腺癌差异表达基因分析^[9]^

基因差异表达分析采用dchip软件分析包进行dchip由哈佛大学生物统计系Cheng LI等联合开发，是综合性芯片分析软件。该软件运行在于windows平台，主要分析Affymetrix基因表达谱及SNP芯片数据，dchip可进行差异基因识别、方差分析、主成分分析、时间序列分析、层次聚类、连锁分析及SNP的拷贝数分析等。我们对GSE10072和GSE7670数据集中质量合格芯片样本分别采用dchip进行差异基因分析，具体操作方法按dchip操作指南进行（http://www.dchip.org），2-fold change的基因被选择为差异表达基因。最后，采用交集方法获得共同差异基因。

#### 1.2.3 文献挖掘方法挖掘已知肺腺癌疾病基因

Genecards（http://www.genecards.org/）是一个收集并展示人类基因及其产物和相关疾病等综合信息的知识平台。它是由以色列的Weizmann研究所基因组研究中心和生物信息学中心共同开发的，含有46 560个基因资料（2.38版），其中24 824个已经被HUGO基因命名委员会审核通过。我们以“lung adenocarcinoma”或“adenocarcinoma of lung”作为搜索词，进入Genecards搜索已知肺腺癌疾病基因^[10]^。同时，也采用Fable文献挖掘工具搜索已知肺腺癌疾病基因，Fable登陆方式：http://www.fable.chop.edu/。

#### 1.2.4 Toppgene筛选新的肺腺癌疾病基因^[11]^

Toppgene（http://toppgene.cchmc.org/）是个有效而方便的基于基因功能相似性的候选基因筛选方法。我们以Genecards搜索到的已知肺腺癌疾病基因作为“training gene set”，而以来自dchip所获得的差异基因作为“test gene set”，然后按Toppgene操作方法获得候选基因。

#### 1.2.5 荧光定量RT-PCR（ΔΔCT法）

收集对数生长期A549或16HBE细胞，按文献方法^[12-14]^分别进行RNA抽提、逆转录及荧光定量PCR反应。反应体系总体积50 μL，由5×SYBR Green I PCR buffer（10 μL）、10 pmol/μL引物F或R（1 μL）、10 mM dNTPs（1 μL）、3 U/μL Taq酶（1 μL）、cDNA（5 μL）及ddH_2_O（31 μL）构成，以β-actin为内参。反应条件设定为：93 ℃、3 min，然后93 ℃、30 s，55 ℃、45 s，72 ℃、45 s，共40个循环。引物设计与合成利用Primer Premier 5.0软件设计特异性引物，使上下游引物跨越1个内含子，由中山大学达安基因公司合成。设计引物序列：CD36（扩增片段长度104 bp）：5’-CAGATGCAGCCTCATTTCCA-3’（Forward Primer），5 ’-AACGTCGGATTCAAATACAGCA-3’（Reverse Primer）；PMAIP1（扩增片段长度79 bp）：5’-GCTCCAGCAGAG CTGGAAGT-3’（Forward Primer），5’-GAAGTTTCTG CCGGAAGTTCAG-3’（Reverse Primer）；FABP4（扩增片段长度106 bp）：5’-GGCATGGCCAAACCTAACAT-3’（Forward Primer），5’-CCTGGCCCAGTATGAAGGAA A-3’（Reverse Primer）；β-actin（扩增片段长度106 bp）（内参基因）：5’-GCATGGGTCAGAAGGATTCCT-3’（Forward Primer），5’-TCGTCCCAGTTGGTGACGAT-3’（Reverse Primer）。

#### 1.2.6 荧光定量PCR数据处理

荧光定量PCR实验数据应用2^-△△Ct^进行处理，其前提是目的基因和内参基因扩增效率相似^[13]^。计算各样本平均CT值和△CT值（Ct=Ct_satb1_-Ct_β-actin_），计算2^-△△Ct^（Ct=Ct_目的样本_-Ct_参照样本_），其数值用于表示目的值相对于参照值的相对倍数。

## 2 结果

### 2.1 肺腺癌差异表达基因

为了获得肺腺癌共同差异表达基因，我们采用dchip分析软件包分别对GSE10072和GSE7670数据集中合格芯片样本进行差异基因分析，最终获得共同差异表达基因344个，其中上调基因94个，下调基因285个（表 1）。

**1:** GSE7670和GSE10072中芯片样本差异表达基因分析结果 Analysis of lung adenocarcinoma differential expression genes against two GEO gene sets GSE10072 and GSE7670

GEO datasets	Platform	Up-expressed gene	Down-expressed gene
GSE7670	GPL96	123	385
GSE10072	GPL96	198	363
Co-expressed gene		94	285

### 2.2 Genecards获得已知肺腺癌疾病基因

以“ l ung adenocarcinoma”或“adenocarcinoma of lung”作为搜索词，进入Genecards搜索已知肺腺癌疾病基因，共获取230条gene card记录；“lung adenocarcinoma”作为搜索词，通过Fable获得118个基因与肺腺癌相关（过滤*n* < 10的基因）。对两种方法获得的疾病基因进行交集分析，浏览每一条文献，过滤不相关的基因，最终获得277个已知肺腺癌疾病基因。

### 2.3 筛选新的肺腺癌疾病基因

采用Toppgene候选基因筛选方法，共获得36个候选疾病基因，经过文献分析，15个基因已有在肺癌方面的报道（各基因报道文献均不多），而另21个基因则在肿瘤方面的研究几无报道（表 2中加下划线基因）。而对21个基因进行KEGG通路富集分析，发现有3个基因（*CD36*、*COL1A1*、*COL3A1*）与ECM-receptor interaction（hsa04512）有关，3个基因（*CSF3*、*CXCL2*、*LEPR*）与cytokine-cytokine receptor interaction（hsa04060）有关，而3个基因（*EDN1*、*EDNRB*、*LEPR*）与neuroactive ligand-receptor interaction（hsa04080）相关。

**2:** Toppgene筛选新的肺腺癌疾病候选基因（注：选取*P* < 0.01的基因） The screen of lung adenocarcinoma candidate genes using Toppgene (Note: Genes were selected based on *P* < 0.01)

Rank	Gene Symbol	Gene ID	Average score	*P*
1	*CD36*	948	0.470 761 5	0.000 000 1
2	*HBEGF*	1839	0.529 077 5	0.000 000 6
3	*PMAIP1*	5366	0.572 612 2	0.000 001 1
4	*TYMS*	7298	0.485 545 3	0.000 001 8
5	*TEK*	7010	0.486 797 7	0.000 001 8
6	*COL1A1*	1277	0.319 382 1	0.000 004
7	I*GFBP3*	3486	0.456 963 2	0.000 006 9
8	*SPP1*	6696	0.503 422 5	0.000 007
9	*EDN1*	1906	0.471 195 9	0.000 008 1
10	*TIE1*	7075	0.541 537 4	0.000 010 4
11	*TGFBR3*	7049	0.497 567 5	0.000 010 4
12	*CDKN1C*	1028	0.431 009 4	0.000 013 1
13	*NR4A1*	3164	0.401 529 2	0.000 017 5
14	*CXCL2*	2920	0.455 321 7	0.000 030 6
15	*BMP2*	650	0.380 468 4	0.000 039 1
16	*CSF3*	1440	0.438 977 1	0.000 200 2
17	*SFN*	2810	0.455 187 2	0.000 266 3
18	*MMP7*	4316	0.398 214 1	0.000 267
19	*COL1A2*	1278	0.362 644 5	0.000 572 2
20	*FABP4*	2167	0.330 210 4	0.000 708 5
21	*CEACAM1*	634	0.404 989 1	0.000 729 4
22	*LEPR*	3953	0.370 081 7	0.001 032 8
23	*DST*	667	0.388 865 5	0.001 492 4
24	*UBE2C*	11065	0.345 610 4	0.001 553 9
25	*GATA6*	2627	0.280 920 4	0.001 912 6
26	*ATF3*	467	0.307 962 8	0.002 073 1
27	*SORBS1*	10580	0.395 258 5	0.002 167 8
28	*CXCL3*	2921	0.384 477 2	0.002 625 5
29	*CRYAB*	1410	0.352 635 4	0.002 788 1
30	*EDNRB*	1910	0.334 035 3	0.003 291 3
31	*DLC1*	10395	0.407 277 2	0.003 520 2
32	*HBB*	3043	0.283 212 9	0.004 263 6
33	*CP*	1356	0.349 502 5	0.004 849 8
34	*COL3A1*	1281	0.291 960 6	0.006 152 1
35	*SLC2A1*	6513	0.263 899 8	0.006 28
36	*NME1*	4830	0.338 435 6	0.010 138 1

### 2.4 荧光定量PCR实验验证

为了验证Toppgene所筛选的基因，我们挑选*CD36*、*PMAIP1*及*FABP4*三个基因，采用荧光定量PCR进行验证，结果表明，与对照组相比，C*D36*、*PMAIP1*及*FABP4*在A549细胞中均为下调表达，此与芯片数据一致（表 3）。

**3:** *CD36*、*PMAIP1*及*FABP4*的荧光定量PCR实验结果 Expression of three genes *CD36*, *PMAIP1* and *FABP4* using fluorescent quantitation PCR

Gene	Sample	Average Ct	Average△CT	Average △△CT	2^-△△Ct^
*CD36*	16-HBE	31.41	13.43	0	1
*CD36*	A549	32.21	13.73	0.285	0.821
*PMAIP1*	16-HBE	34.34	16.36	0	1
*PMAIP1*	A549	40.96	22.48	7.12	0.0072
*FABP4*	16-HBE	31.24	13.26	0	1
*FABP4*	A549	32.51	14.03	0.77	0.588
*β-actin*	16-HBE	17.98			
*β-actin*	A549	18.48			
△Ct=target gene Ct-actin Ct; △△Ct=sample target gene△Ct-reference sample target gene△Ct; when the amplification rate of PCR get close to 100%, relative sample template product=2^-△△Ct^.					

## 3 讨论

当前，基因连锁和基因表达谱分析等高通量基因组分析方法能有效地对基因进行分类，并产生数百个候选疾病基因，但不能提供足够的疾病特异性基因信息，因此，这些方法在疾病基因发现方面存在较大问题^[15]^。近年来，生物信息学方法广泛应用于疾病基因发现，特别是ToppGene在疾病基因发现方面具有独特点。本研究中，我们的兴趣在于通过计算生物学策略“ToppGene”，发现新的肺腺癌疾病基因。通过本研究，我们筛选到36个候选疾病基因，经过文献分析，发现21个基因在肿瘤方面的研究几无报道（Pubmed数库范围内）。随后，我们选取*CD36*、*PMAIP1*及*FABP4*三个基因进行荧光定量PCR验证，结果发现*CD36*、*PMAIP1*及*FABP4*在A549细胞中均下调表达，与芯片数据相一致。

随着生物技术的快速发展，生物信息量也成爆炸式增长，生物医学文献作为成果展示和学术交流的主要方式之一，其数目之大、增长速度之快远远超过了其它学科领域，例如，Medline收集了全世界4 800多种生物学及医学杂志上的1 800多万篇文献，并且以每个月超过万篇的速度增长。海量的文献中蕴涵着丰富的生物学信息，因此，如何挖掘和发现其中有生物学意义的信息具有重要意义。Genecards^[10]^是一种收载较为全面的基因数据平台，对基因注释全面而规范；Fable也是一种功能强大的文献挖掘工具，特别是在人类疾病基因和蛋白的挖掘方面功能具有独特优势。为了更全面地确定已知肺腺癌疾病基因，在本研究中，我们联合应用Genecards和Fable两种文献挖掘工具，建立一个含277个基因的“训练基因集”，并应用此“训练基因集”最终筛选到肺腺癌候选疾病基因。

Toppgene^[11]^是一种基于功能相似性的候选疾病基因筛选工具，Toppgene最大优点在于，它可从GO注释、通路、蛋白相互作用、疾病表型、疾病、转录因子等14个方面对候选基因进行全面评估，最后依据总体*P*值对候选基因进行排序。与其它基于功能相似性的候选基因发现方法一样，基于Toppgene的候选疾病基因筛选方面也有一定的缺陷，如：①仍有约1/3的基因没有作功能注释；②仅有部分的基因具有通路和表型注释；③蛋白质相互作用数据仍不完善，特别是通过实验验证的数据有限。相信，随着生物信息学与各种生物技术的快速发展，Toppgene获得的结果会越来越完善。

总之，通过本研究，我们筛选到一些可供进一步实验研究的肺腺癌候选基因，有关这此候选基因在肺腺癌发生发展中的作用仍需进一步的实验证实。
